# Effect of Protein Denaturation and Enzyme Inhibitors on Proteasomal-Mediated Production of Peptides in Human Embryonic Kidney Cells

**DOI:** 10.3390/biom9060207

**Published:** 2019-05-28

**Authors:** Sayani Dasgupta, Michael A. Fishman, Leandro M. Castro, Alexandre K. Tashima, Emer S. Ferro, Lloyd D. Fricker

**Affiliations:** 1 Department of Molecular Pharmacology, Albert Einstein College of Medicine, Bronx, NY 10461, USA; sayani.dasgupta@einstein.yu.edu (S.D.); michael.fishman@einstein.yu.edu (M.A.F.); 2 Bioscience Institute, Coastal Campus, São Paulo State University, 11330-900 São Vicente, SP, Brazil; leandro.mantovani@unesp.br; 3 Department of Biochemistry, Escola Paulista de Medicina, Federal University of Sao Paulo, 04023-901 Sao Paulo, SP, Brazil; aktashima@unifesp.br; 4 Department of Pharmacology, Biomedical Science Institute, University of São Paulo, 05508-000 São Paulo, SP, Brazil; 5 Department of Neuroscience, Albert Einstein College of Medicine, Bronx, NY 10461, USA

**Keywords:** Proteasome, peptide, peptidomics, mass spectrometry, bortezomib, epoxomicin

## Abstract

Peptides produced by the proteasome have been proposed to function as signaling molecules that regulate a number of biological processes. In the current study, we used quantitative peptidomics to test whether conditions that affect protein stability, synthesis, or turnover cause changes in the levels of peptides in Human Embryonic Kidney 293T (HEK293T) cells. Mild heat shock (42 °C for 1 h) or treatment with the deubiquitinase inhibitor b-AP15 led to higher levels of ubiquitinated proteins but did not significantly increase the levels of intracellular peptides. Treatment with cycloheximide, an inhibitor of protein translation, did not substantially alter the levels of intracellular peptides identified herein. Cells treated with a combination of epoxomicin and bortezomib showed large increases in the levels of most peptides, relative to the levels in cells treated with either compound alone. Taken together with previous studies, these results support a mechanism in which the proteasome cleaves proteins into peptides that are readily detected in our assays (i.e., 6–37 amino acids) and then further degrades many of these peptides into smaller fragments.

## 1. Introduction

Mass spectrometry-based quantitative peptidomic techniques were initially developed to identify neuropeptides and peptide hormones in a variety of organisms [1,2,3,4]. In addition to detecting a large number of neuropeptides and other peptides derived from secretory pathway proteins, peptidomic studies detected hundreds of peptides derived from cytosolic, nuclear, and mitochondrial proteins [5]. Some of these intracellular peptides may play a role in biological functions by regulating protein–protein interactions, much like synthetic peptides modulate cellular functions by mimicking or blocking protein–protein interactions [5,6,7]. Endogenous peptides generated from mitochondrial proteins by the matrix-localized protease ClpP have been shown to signal unfolded protein response and activate mitochondrial chaperone genes in *C. elegans* [8]. In *Drosophila*, peptides encoded by short open reading frames of *pri* RNA affect transcription by mediating proteasomal processing of *Svb* transcriptional repressor into a shorter activator [9]. Taken together, these studies suggest that intracellular peptides play important roles in regulating cellular functions.

Proteasomes are the major peptide-producing enzyme complexes within cells [10,11,12]. All proteasome forms contain a 20S catalytic core that contains two outer α rings and two inner β rings, each containing four catalytically inactive subunits (β3, β4, β6 and β7) and three proteolytic subunits: β-1 (alternatively referred to as ‘caspase-like’ or ‘peptidylglutamyl peptide hydrolyzing’), β-2 (referred to as ‘trypsin-like’), and β-5 (referred to as ‘chymotrypsin-like’). The 20S core exists alone within the cell and in complex with various regulatory caps on one or both ends of the 20S core, producing a large number of distinct forms [13,14]. A major form is the 26S proteasome, which is composed of two 19S regulatory caps surrounding the 20S catalytic core [14,15]. Proteins targeted for degradation by the covalent addition of ubiquitin are recognized by the 19S regulatory particle, ubiquitin is removed by proteasome-associated deubiquitinases (DUBs), and the protein is unfolded in an ATP-dependent process and translocated into the catalytic 20S core [16]. The 20S catalytic core can associate with two other families of regulatory particles, PA200 and PA28 (also known as 11S), which form complexes that cleave small intrinsically unfolded proteins in a process that does not require ATP or ubiquitin [13,14]. The 26S proteasome and the 20S catalytic core may also hydrolyze intrinsically disordered proteins in a ubiquitin-independent manner. 

Most of the peptides produced by the proteasome are rapidly degraded within seconds by intracellular peptidases, based on studies using a small number of synthetic peptides [17,18,19]. However, some of these synthetic peptides are more stable, with half-lives considerably longer than that of the average peptide [17,18,19]. Hundreds of endogenous peptides have been detected in cell and tissue extracts using mass spectrometry-based techniques [5,6,20]. Because the levels of most of these peptides are greatly reduced by the treatment of cells with epoxomicin, these peptides are presumably products of proteasome-mediated protein cleavages [21]. The observed peptides are generally within the size range that is known to be produced by the proteasome, which is typically said to be 3–22 residues—this size range is based on studies that investigated the degradation of several denatured, nonubiquitinated proteins by purified 20S and 26S proteasomes [22]. However, the proteasome is also able to cleave proteins and release fragments much larger than 22 residues, including NF-κB (nuclear factor kappa-light-chain-enhancer of activated B cells) and other proteins [23,24]. Furthermore, intracellular peptides of up to 37 amino acids are greatly decreased by the treatment of cells with epoxomicin, suggesting that proteasome-mediated cleavages can produce peptides larger than 22 residues [21]. 

The intracellular peptides detected in the peptidomics studies commonly have hydrophobic residues in the P1 site, primarily Leu, Val, Phe, and Tyr, which is consistent with the major role of the β5 chymotrypsin-like proteasome subunit in the cleavage of proteins [20,21,25,26,27,28]. Unexpectedly, 30–50% of the peptides observed in peptidomics studies represent the N- or C-terminal fragments of the proteins [20]. In contrast, when proteins are digested in vitro with proteasomes or endopeptidases such as trypsin, nearly all of the products are internal fragments and there is only a single N- and C-terminal fragment [22,29,30]. A related issue is that the peptides detected in cell/tissue extracts are derived from a small number of abundant cellular proteins that are considerably smaller than the average protein [20,31]. This is also unexpected because larger proteins should be more highly represented based on the greater number of potential peptides that should be generated upon digestion. Thus, the cellular peptidome represents a small fraction of the potential fragments of a subset of the major cellular proteins.

It is possible that at least part of the cellular peptidome is derived from unfolded or denatured proteins that are preferentially degraded by some forms of the proteasome [14,32]. To test this, we used a quantitative peptidomics method to detect and measure the levels of peptides in HEK293T cells in response to heat shock, a condition that increases unfolded proteins. We also tested whether an inhibitor of proteasome-associated DUBs affected the levels of peptides; b-AP15 inhibits the degradation of ubiquitin-dependent proteasome substrates but not of ubiquitin-independent proteasome substrates [33,34]. Some previous studies on major histocompatibility complex (MHC) class I-bound peptides reported that they are preferentially produced from newly synthesized proteins and from defective ribosomal products, based on sensitivity to protein translation inhibitors such as cycloheximide [35,36]. To address this possibility, we tested whether cycloheximide treatment altered the levels of intracellular peptides. We also tested the effect of combinations of bortezomib and epoxomicin based on the paradoxical findings that epoxomicin reduced the levels of most cellular peptides [21] while bortezomib elevated the levels of many peptides [26,27]. Although bortezomib has been reported to have off-target effects on other cellular peptidases [26,37], the inhibitors of major cellular peptidases such as aminopeptidases and tripeptidylpeptidase 2 had no discernible effect on the levels of intracellular peptides [26]. In addition, the levels of most cellular peptides are not altered in response to elevated intracellular calcium, suggesting that calpain is not responsible for their production [20,31]. Together with previous studies, the results of the present study point to a complex mechanism by which proteasomes generate and degrade intracellular peptides.

## 2. Results

**Figure 1:**
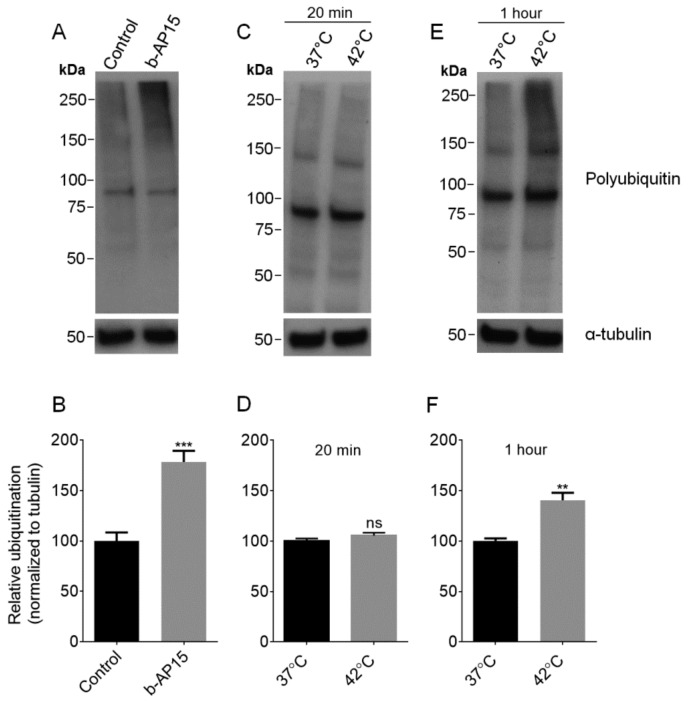
Analysis of the effect of b-AP15 and heat shock on the ubiquitination of proteins, using anti-ubiquitin antibody. Top panels: representative Western blots for ubiquitination and α-tubulin (as loading control) in HEK 293T cells after various treatments. Lower panels: the broad “smear” of ubiquitinated proteins from 50 kDa to the top of the gel was quantified using ImageJ and normalized to α-tubulin. Treatments: 1 µM b-AP15 in 0.05% DMSO for 1 h or control (0.05% DMSO alone) for 1 h (**A**,**B**); heat shock at 42 or 37 °C control for 20 min (**C**,**D**); heat shock at 42 or 37 °C control for 1 h (**E**,**F**). Error bars represent the standard error of mean (*n* = 6). ***, *p* ≤ 0.001 and **, *p* ≤ 0.01 using Student’s *t*-test; ns: not significant.

Many proteins are targeted for degradation by the addition of ubiquitin, and the ubiquitin must be removed by proteasome-associated DUBs before the protein can enter into the inner degradation chamber of the proteasome [33,34]. To investigate whether this pathway is required for the production of intracellular peptides, we treated cells with b-AP15 which inhibits two of the DUBs that are transiently associated with the 19S regulatory particle: ubiquitin C-terminal hydrolase 5 (UCHL5) and ubiquitin-specific peptidase 14 (USP14) [32,34]. To confirm that b-AP15 is able to block deubiquitination in our system, we treated HEK293T cells with either 1 μM b-AP15 or a comparable concentration of DMSO for 1 h and probed the cell lysate with an antibody that detects ubiquitin, polyubiquitin, and ubiquitinated proteins. Cells treated with b-AP15 have elevated the levels of high-molecular-weight polyubiquitinated proteins (Figure 1A). Densitometric analysis of the high-molecular-weight smear with ImageJ showed that b-AP15 significantly increased protein ubiquitination by approximately 80% (Figure 1B). 

To investigate whether b-AP15 affects the levels of intracellular peptides, HEK293T cells were treated with either 1 μM b-AP15 or a comparable concentration of DMSO for 1 h and the relative levels of cellular peptides were measured using a quantitative peptidomics approach. In this experiment, two biological replicates of the treated samples and two biological replicates of the control cells were analyzed using a total of four distinct isotopic tags; this allowed the comparison of biological replicates of the treated and control cells in the same liquid chromatography-mass spectrometry (LC–MS) run (Figure S1). A total of 152 distinct peptides, representing naturally occurring fragments from 57 proteins, were identified by MS/MS sequence analysis, and 150 additional peptides were detected that could not be identified from MS/MS but which could be quantified from the MS spectra. The entire data set is shown in Table S1, with each row representing a peptide found in one of the LC–MS runs (note that there are 275 rows in Table S1 for the b-AP15 data set, but because some peptides were found in both LC–MS runs, these 275 rows represent only 152 distinct peptides).

**Figure 2:**
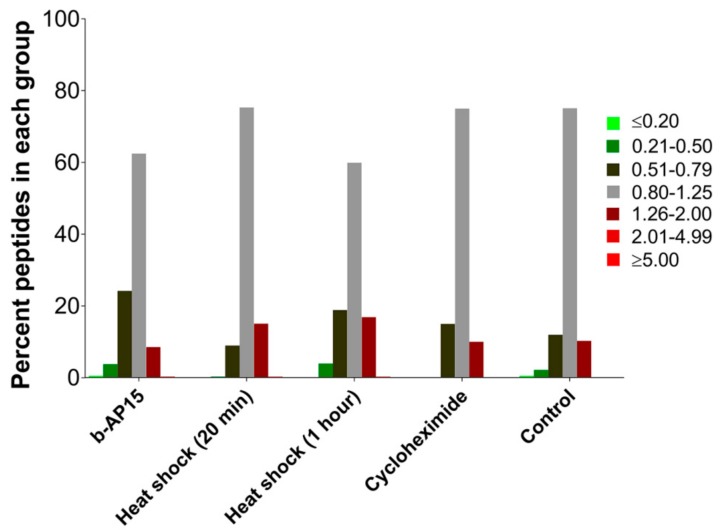
Percentage of peptides present in groups divided according to relative level. For samples subjected to b-AP15 (1 µM, 1 h), heat shock (42 °C for 20 min and 1 h), and cycloheximide (0.2 mM, 35 min) treatment, the ratios for both replicates were averaged for the analysis and the peptides were subsequently grouped based on the average relative levels. For peptides found multiple times with different charge states and/or numbers of tags, the peak intensities were summed and expressed as the ratio of average control such that a single value for peptide ratio could be obtained for each replicate, which were then averaged. For the controls, peptide ratios of each replicate of every peptide from all the experiments were combined together for the analysis. Data for each of the peptides are provided in Table S1.

To visualize whether the treatment caused a change in the relative levels of the identified peptides, the results were divided into seven groups: decreased ≥5-fold (i.e., ratio of treated/control ≤0.20); decreased between 2–5-fold (ratio 0.21–0.50); slightly decreased (ratio 0.51 to 0.79); not greatly affected (ratio 0.80–1.25); slightly increased (ratio 1.26–2.0); increased between 2–5-fold (ratio 2.01–4.99); and increased ≥5-fold (ratio ≥5.0). The ratios 0.80 and 1.25 were based on 4/5 and 5/4, respectively. Previous studies using the quantitative peptidomic technique found that the majority of peptides in the control cells fell within the range of 0.80 to 1.25 [25,26,31]. Likewise, in the present study, ~75% of the peptides in the control cells (i.e., the C1 and C2 data in Table S1) fell into this range, and a minority of peptides fell into the “slightly” increased and decreased groups (Figure 2); this reflects normal biological variation. Following treatment with b-AP15, the majority of peptides (63%) were also in this range, indicating that there was no major effect on these levels of these peptides (Figure 2).

**Figure 3:**
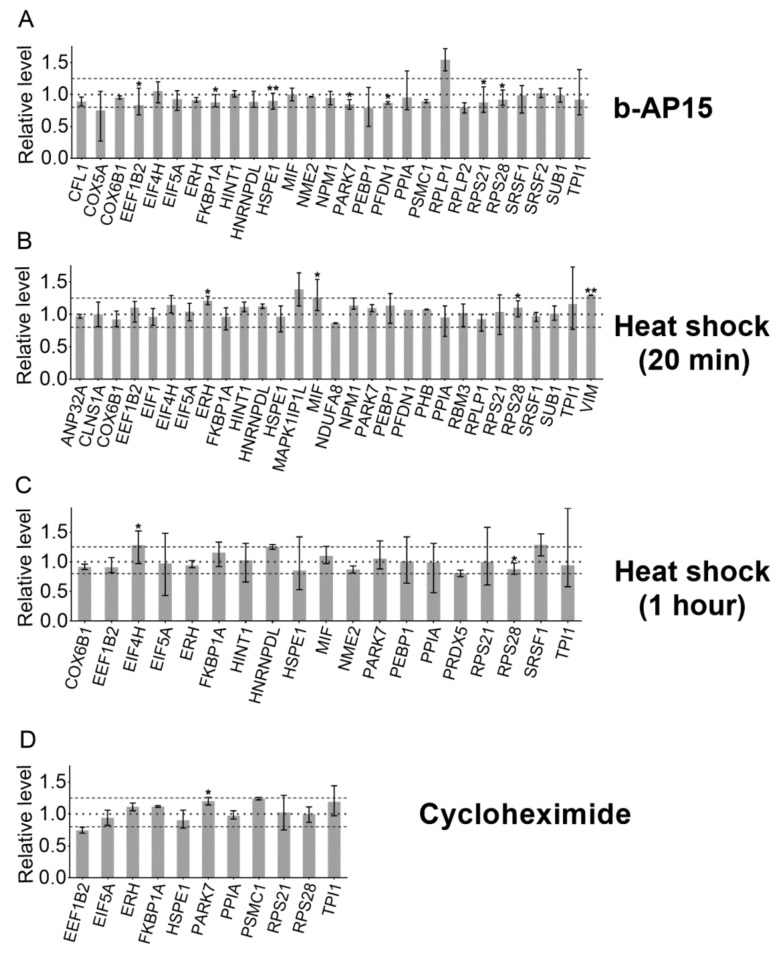
Mean relative levels of peptides from proteins in response to treatment with 1 µM b-AP15 for 1 h (**A**), 42 °C heat shock for 20 min (**B**) or 1 h (**C**), and 0.2 mM cycloheximide for 35 min (**D**). For all the proteins that gave rise to at least two peptides, the relative levels of all peptides (including different charge states and/or number of tags) derived from a single protein were averaged. Ratios of 0.8 and 1.25 are marked with dashed lines, and 1.0 is marked with a dotted line. Error bars indicate the range between the largest (upwards error bar) and smallest (downwards error bar) peptide ratio from each protein. * *p* < 0.05; ** *p* < 0.01. Mean relative levels of peptides derived from these proteins, statistical significance, and number of peptides from each protein are provided in Table S2.

To assess this more carefully, we focused on only the identified peptides and combined data for peptides derived from the same protein; 27 proteins detected in this analysis were represented by two or more peptides. The relative levels of all peptides derived from a single protein were averaged and plotted, with error bars showing the range of the values for distinct peptides (Figure 3). For the majority of the proteins, the average levels of peptides in the b-AP15-treated samples were not statistically different than their levels in the DMSO controls (Table S2). Thus, the inhibition of deubiquitination by b-AP15 does not alter the formation of the major intracellular peptides, indicating that the DUBs targeted by b-AP15 (USP14 and UCHL5) are not required for the production of the observed peptides. These results do not rule out the possibility that other major DUBs such as RPN11 contribute.

Some forms of the proteasome preferentially cleave proteins that are partially unfolded [16]. If the majority of the intracellular peptides are derived from partially unfolded proteins, we hypothesized that the levels of peptides would be increased by brief heat shock. To test this, cells were incubated at 42 °C for either 20 min or 1 h, and the respective control cells were allowed to remain at 37 °C. Previously, heat shock with 42 or 45 °C has been shown to induce protein unfolding and increase the levels of ubiquitinated proteins [38,39]. The level of ubiquitinated proteins was elevated ~40% by 1-h treatment at 42 °C, but not significantly affected after only 20 min (Figure 1C–F). Peptides were extracted from the heat-treated cells and the control cells were incubated at 37 °C and the relative levels were determined using quantitative peptidomics (labeling scheme shown in Figure S1). A total of 156 peptides, derived from 61 proteins, were identified by MS/MS sequence analysis for the 20-min heat shock experiment, and 151 peptides from 62 proteins were identified for the 1-h heat shock experiment (Table S1). The levels of the majority of peptides were not greatly altered with heat shock for either 20 min or 1 h (Figure 2). Data for identified peptides derived from the same proteins were combined and the average levels were plotted (Figure 3). For the majority of the proteins, the average levels of peptides in the heat-treated samples were not statistically different than their levels in the controls (Table S2). These results suggest that brief the exposure of cells to elevated temperatures does not lead to an increase in intracellular peptides.

Previous studies found that the levels of some MHC class I-bound peptides are reduced by treatment with protein translation inhibitors such as cycloheximide, suggesting that these peptides are preferentially produced from newly synthesized proteins [40]. To investigate whether the major intracellular peptides detected in our studies are derived from newly synthesized proteins, cells were treated for 35 min with 0.2 mM cycloheximide or medium alone and assayed using the quantitative peptidomics approach (labeling scheme shown in Figure S1). This concentration of cycloheximide and length of treatment were previously shown to greatly alter the levels of newly synthesized proteins [40]. Fifty peptides derived from 28 proteins were identified by MS/MS analysis (Table S1). As a side point, the detection of fewer peptides in this experiment (compared to the other experiments) was not due to the cycloheximide treatment; fewer peptides were found in both the treated and the untreated control cells in this experiment. Some LC/MS runs yield fewer peptides than other runs, depending on the state of the mass spectrometer at the time of analysis and other variables between experiments. The levels of peptides in the cycloheximide-treated samples were generally comparable to those in the control cells (Figure 2). Of the 11 proteins found with two or more peptides, the average levels of peptides from most of these proteins in the cycloheximide-treated samples were not statistically different than their levels in the controls (Figure 3, Table S2). Taken together, these results suggest that the intracellular peptides detected herein are not derived from newly synthesized precursors.

### Effect of Combinations of Proteasome Inhibitors on the Levels of Intracellular Peptides

**Figure 4:**
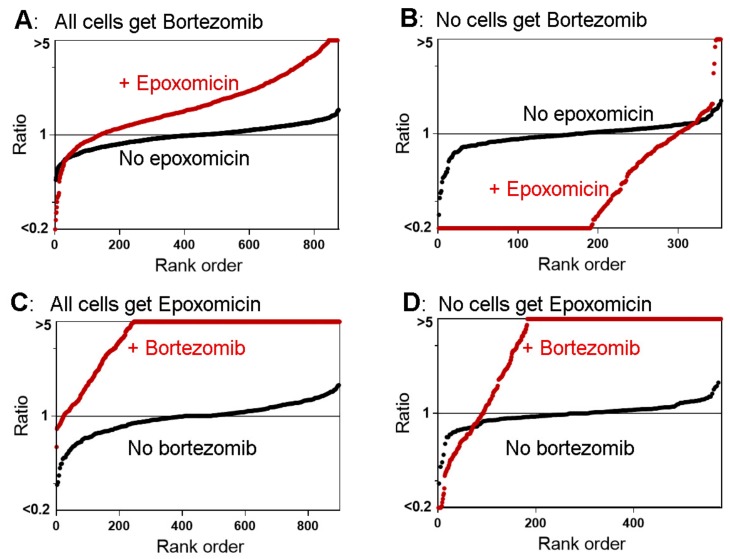
Summary plots of the peptidome of HEK293T cells in response to proteasome inhibitors. (**A**) Cells were treated for 30 min with a combination of 0.2 µM epoxomicin and 0.5 µM bortezomib or with 0.5 µM bortezomib alone (all in the presence of 0.05% DMSO). (**B**) Data from a previously published study in which cells were treated with 0.2 µM epoxomicin in 0.05% DMSO, or 0.05% DMSO alone for 1 h [21]. (**C**) Cells were treated for 30 min with a combination of 0.2 µM epoxomicin and 0.5 µM bortezomib or with 0.2 µM epoxomicin alone; all samples included 0.05% DMSO. (**D**) Data from a previously published study in which cells were treated with 0.5 µM bortezomib in 0.05% DMSO or with 0.05% DMSO for 30 min [27]. The y-axis represents the relative levels of peptides (log-scale) and the x-axis represents the rank order of peptides sorted according to the relative level. If the ratio was <0.20 or >5.0, the value was capped at 0.20 or 5.0 to reflect the typical signal-to-noise ratio. Red circles indicate the ratio of each replicate of identified peptides in cells treated with the combination of epoxomicin and bortezomib in panels A and C and either inhibitor alone in panels B and D, expressed relative to the average of the respective controls (i.e., for panel A, the red circles represent the relative level of each peptide in cells treated with both inhibitors relative to cells treated with bortezomib alone, while, for panel C, the red circles represent the relative level of each peptide in cells treated with both inhibitors relative to cells treated with epoxomicin alone). Black circles indicate the ratio of each control replicate expressed relative to the average control value (either bortezomib or epoxomicin alone in panels A and C; DMSO in panels B and D). See Figure S2 for details on the treatments of cells for each of these peptidomic studies.

In previous peptidomic studies on cell lines, short-term treatment with 0.2 μM epoxomicin produced a decrease in the levels of the vast majority of peptides, whereas 0.05 and 0.5 μM bortezomib produced a decrease of some peptides but paradoxically elevated the levels of most peptides, including many which are the products of cleavages at hydrophobic sites; these were predicted to decrease because bortezomib inhibits the β5 proteasome subunit [21,26,27]. A number of other proteasome inhibitors were also examined for their effect on the peptidome and were found to produce results that were either similar to epoxomicin (MG132, lactacystin), similar to bortezomib (MG262), or intermediate in their effect (carfilzomib, MLN2238) [26]. To further explore the paradoxical finding, we treated HEK293T cells with combinations of epoxomicin and bortezomib. For these studies, “control” cells were treated with either inhibitor alone (Figure S2), so that the effect of the combination could be directly compared to the individual inhibitors. A total of 250 peptides derived from 57 proteins were identified in the experiment comparing the combination versus bortezomib alone, and 249 peptides derived from 62 proteins were identified in the experiment comparing the combination to epoxomicin alone (Table S3). To visualize the relative levels of all the identified peptides, rank-order plots were generated in which the relative value of every peptide in each replicate was sorted from low to high and plotted with the y-axis representing the relative ratio and the x-axis the rank order (Figure 4). If the ratio was <0.20 or >5.0, the value was capped at 0.20 or 5.0 to reflect the typical signal-to-noise ratio; in some cases, the signal-to-noise ratio was greater than 5:1 but it was capped at this value for uniformity. When compared to bortezomib alone, the combination of epoxomicin and bortezomib moderately increased the levels of most peptides (Figure 4A). This finding was unexpected because epoxomicin was previously found to decrease the levels of most peptides; data from a previous study examining the effect of 0.2 μM epoxomicin alone [21] were plotted for comparison (Figure 4B). When compared to epoxomicin alone, the combination of bortezomib and epoxomicin produced a dramatic increase in the levels of most peptides (Figure 4C). This is generally similar to the previous result that bortezomib elevated the levels of many peptides; data from a previous study testing 0.5 μM bortezomib alone [26] were included for comparison (Figure 4D).

**Figure 5:**
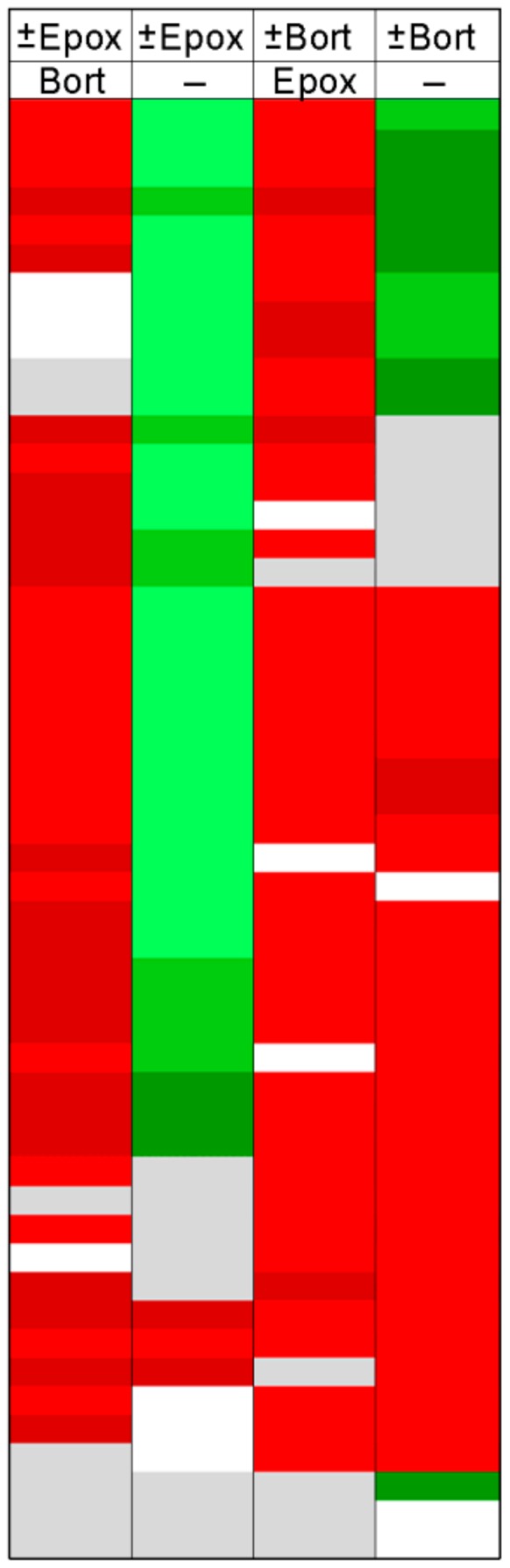
Heat map analysis of selected peptides. Peptides commonly detected in most experiments were selected for this analysis. Each row denotes a specific peptide, and each column represents a different experiment described in Figure 4. Peak intensities of peptides found with multiple charge states and/or numbers of tags were summed and expressed as a ratio of average control such that a single value for the peptide ratio could be obtained for each replicate. The ratio was color-coded using the scheme shown in Figure 2, with green representing decreases and red representing increases. Grey represents peptides that did not change substantially. White corresponds to peptides that were either not detected or which could not be accurately quantified due to peak overlap with another co-eluting peptide. Names of protein precursors, peptide sequences, mass, cleavage sites, and peptide ratios are provided in Table S4.

The rank-order analysis does not provide information about specific peptides. To examine whether the same peptides were differentially affected by the various treatments, we created heat maps of peptides that were identified in multiple experiments (Figure 5). For this plot, the most commonly detected peptides found in nearly every experiment were considered. Details of these peptides, such as sequence, mass, cleavage sites, and precursor protein are provided in Table S4 along with quantitative data. Each of the rows represents a different peptide and each column represents an experiment. Peptides showing a very large decrease relative to the average control are in bright green; peptides that greatly increased are in bright red, and peptides showing smaller changes are in gradated shades; gray represents peptides that did not substantially change and white represents peptides either not detected or for which peak overlap precluded analysis. Many of the peptides elevated by epoxomicin in the presence of bortezomib (Figure 5, column 1) were decreased when epoxomicin was compared to the control cells (Figure 5, column 2). In these studies, all cells were treated with the same concentration of DMSO that was used to dissolve the bortezomib and/or epoxomicin, so the difference in levels of peptides is not due to DMSO treatment. The addition of bortezomib to the cells caused many of the same peptides to increase, regardless of whether epoxomicin was included (Figure 5, column 3) or not (column 4).

**Figure 6:**
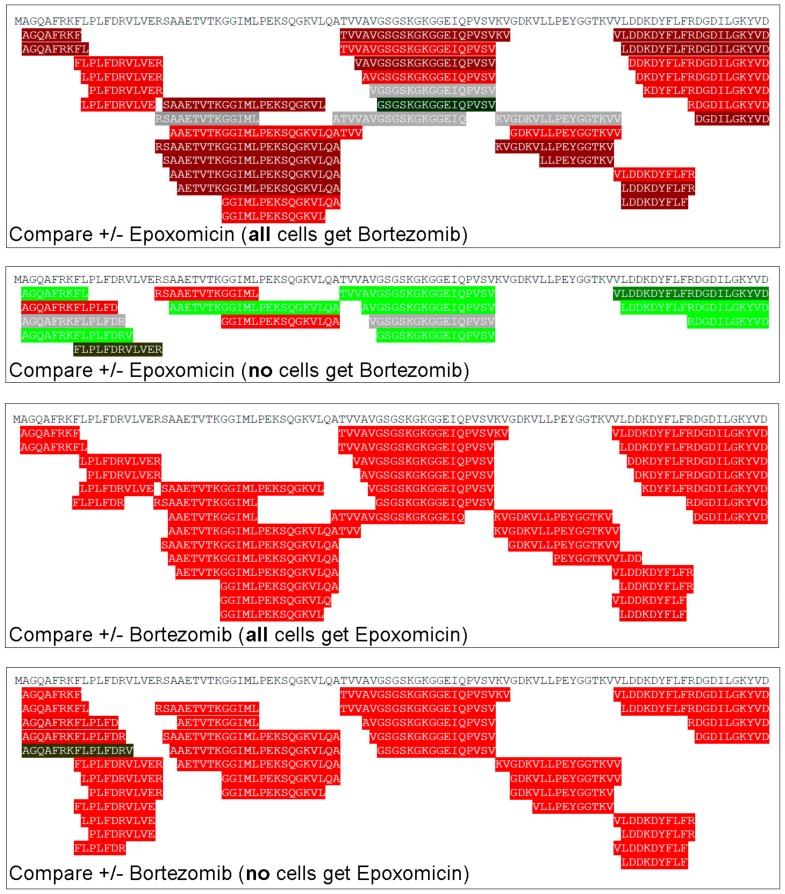
Relative levels of all peptides derived from heat shock 10-kDa protein 1 in HEK293T cells. Cells were treated as described in Figure 4 and the ratio for each peptide was color-coded using the scheme shown in Figure 2, with green representing decreases, red representing increases, and grey representing no substantial change.

To compare peptides that are derived from the same protein, we selected heat shock 10-kDa protein 1 (gene name *HSPE1*) because a large number of peptides were found to be produced from this protein (Figure 6). This analysis is distinct from that of the heat map because all peptides derived from the protein are shown, not just those found in multiple experiments as shown in the heat map. The overall results from the analysis of *HSPE1*-derived peptides is similar to those from the analysis of peptides in the heat map; bortezomib produces a large increase in the levels of nearly all peptides regardless of whether epoxomicin is present, while the effect of epoxomicin is much different in cells treated with bortezomib versus cells without bortezomib (Figure 6). Three *HSPE1*-derived peptides were greatly increased when epoxomicin was tested alone (Figure 6, second panel; red bars). Of these three peptides, one was also increased when epoxomicin was added to cells treated with bortezomib, one was not affected by epoxomicin, and the third was not detected in the experiment comparing the addition of epoxomicin to bortezomib treated cells (Figure 6, top panel). In contrast, nearly all of the peptides found to decrease when the epoxomicin-treated cells were compared to the control cells (Figure 6, second panel; green bars) were elevated when cells treated with a combination of epoxomicin and bortezomib were compared to cells treated with bortezomib alone (top panel; red bars). In the experiments comparing the addition of bortezomib, either to the epoxomicin-treated cells (Figure 6, third panel) or to the control cells (fourth panel), many of the same *HSPE1*-derived peptides were detected and found to be greatly elevated by the addition of bortezomib.

**Figure 7:**
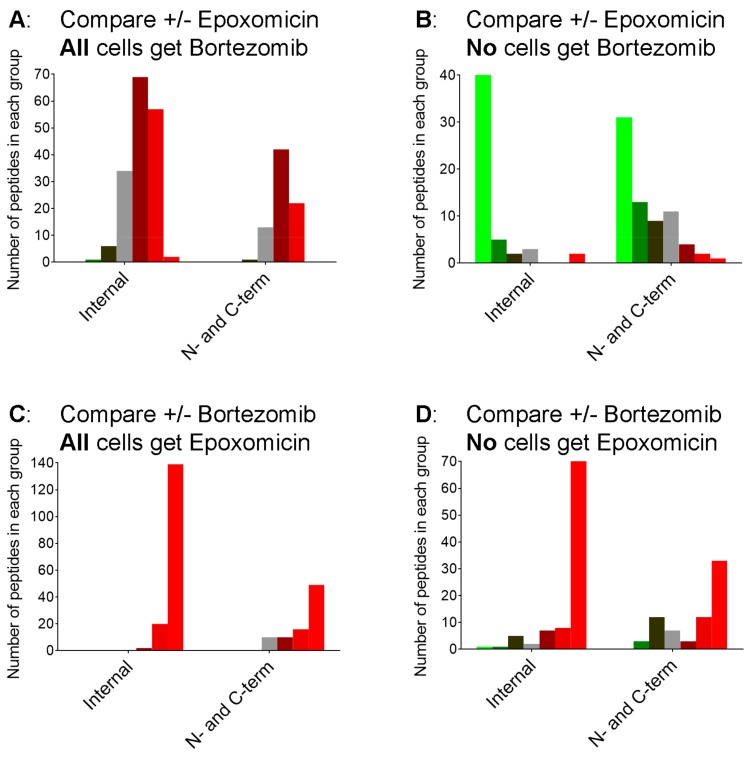
Correlation of the effect of proteasome inhibitors with the location of the peptide within the protein precursor. The N-terminal group includes peptides lacking the initiation Met. Cells were treated as described in Figure 4 and color coded as described in Figure 2.

In previous studies, it was noted that 40–50% of the peptides found in HEK293T and other cell lines, and in mouse brain, represent the N- or C-terminus of the protein [5,20,25,26,27]. It was also noted that the treatment of cells with bortezomib caused an increase mainly in the fraction of peptides that represent internal fragments of their proteins [27]. Therefore, we examined the data from the present study by the location of the peptide within the protein precursor, comparing internal peptides with those derived from the N- or C-terminus of the protein. In the experiment testing the addition of epoxomicin in the presence of bortezomib, both groups of peptides showed a similar response to epoxomicin (Figure 7, panel A). In contrast, the addition of epoxomicin to cells in the absence of bortezomib caused a pronounced decrease in nearly all of the internal peptides but only a subset of the N- and C-terminal peptides (Figure 7, panel B; compare bright green bars versus other bars). The addition of bortezomib to cells treated with epoxomicin caused a large increase in internal peptides (Figure 7, panel C), which was similar to the effect of bortezomib in cells without epoxomicin (Figure 7, panel D).

## 3. Discussion

Peptidomics techniques were originally developed to detect neuropeptides in mouse brain and other organisms [1,2,3,4]. In addition to the detection of neuropeptides and other fragments of secretory pathway proteins, a large number of peptides were found to arise from intracellular proteins [5,41]. Whereas the levels of most neuropeptides were decreased in mice lacking neuropeptide-processing enzymes (i.e., prohormone convertases, carboxypeptidase E), the levels of intracellular peptides in these mice were comparable to the levels in wild-type mice, suggesting that the intracellular peptides are relatively stable [42,43,44,45,46,47]. The treatment of human and mouse cell lines with proteasome inhibitors greatly altered the levels of nearly all of the intracellular peptides, suggesting that the proteasome is involved in their production [21,25,26,27]. This effect was rapid, occurring within 1 h of treatment, indicating that the pool of intracellular peptides is highly dynamic. Recently, a number of peptides derived from intracellular proteins were identified in yeast [31] and zebrafish [48], with many similarities between the peptides in these organisms and those found in different human and mouse cell lines [20,25].

An important step in ubiquitin-dependent proteasome degradation by the 26S proteasome is the removal of ubiquitin from the polyubiquitinated proteins [33,34,49]. This step is performed by DUBs; one DUB (RPN11) is a component of the proteasome and two (USP14, and UCHL5) are transiently associated with the 19S regulatory proteasome cap [32]. The inhibition of USP14 and UCHL5 with b-AP15 leads to elevated levels of ubiquitinated proteins in previous studies [34] and in the present study on HEK293T cells (Figure 1). Despite the elevation of ubiquitinated proteins, b-AP15 treatment does not greatly influence the levels of intracellular peptides (Figure 2 and Figure 3). This finding suggests that the production of intracellular peptides detected in our study does not require the USP14 and UCHL5 deubiquitinases.

Intracellular protein degradation is enhanced by conditions such as heat stress, which increases the levels of denatured proteins [39,50]. Protein degradation of denatured proteins may proceed in part through the ubiquitin-mediated system, as evident from the increase in the levels of ubiquitinated proteins following heat shock in HEK293T cells (Figure 1) and other cell lines [38]. There is also a ubiquitin-independent component of the degradation of misfolded proteins [51,52]. The present finding that heat treatment did not substantially alter the levels of intracellular peptides (Figure 2 and Figure 3) suggests that their production is independent of the unfolded protein response.

The present study was focused on the major intracellular peptides detected in extracts of cells; these peptides are completely distinct from MHC class I-bound peptides [26,27]. However, both sets of peptides are initially produced via proteasome-mediated degradation of cellular proteins, and therefore we considered previous studies that investigated the initial steps in the production of MHC class I-bound peptides. It has been proposed that most MHC class I peptides are generated from proteasomal degradation of defective ribosomal products and/or other newly synthesized proteins based on the finding that the inhibition of protein synthesis by cycloheximide reduces the production of MHC class I peptides [35,36,40,53,54,55,56]. In the present study, the treatment of HEK293T cells with 0.2 mM cycloheximide for 35 min had no major effect on the levels of most intracellular peptides (Figure 2 and Figure 3). Because this concentration of cycloheximide blocks protein synthesis within minutes [57], our results suggests that the intracellular peptides detected in our studies are not derived from newly synthesized proteins.

Previous studies found that the levels of some peptides were greatly reduced by the treatment of cells with proteasome inhibitors, including bortezomib (50 and 500 nM), epoxomicin (0.2 and 2 µM), and five other inhibitors [21,25,26,27]. However, many other peptides were elevated when cells were treated with bortezomib and MG262 [26,27]. Only a small number of peptides were greatly elevated by treatment with epoxomicin, and these were mainly peptides formed by proteasome β1 subunit-mediated cleavages (i.e., at residues with Glu or Asp in the P1 site) [21]. Because epoxomicin potently inhibits the β5 subunit and at higher concentrations also inhibits the β2 subunit, the increase in peptides resulting from cleavage at β1 sites presumably reflects the enhanced role of this subunit when the other two subunits are inhibited by epoxomicin [21]. In contrast, the elevated levels of peptides caused by bortezomib is not easily explained by this mechanism; most of the peptides elevated by bortezomib arise from cleavages at sites which fit the consensus for cleavage by the β5 subunit, which is the main target of bortezomib. Alternative hypotheses can potentially explain this apparent paradox, such as the possibility that bortezomib inhibits a non-proteasomal enzyme that degrades the proteasome-produced peptides. This and other possibilities were considered and discussed in our initial publication describing the paradoxical effect of bortezomib and in a subsequent study [26,27]. The present study further explores this apparent paradox by testing a combination of epoxomicin and bortezomib. Because epoxomicin is an irreversible inhibitor, it was predicted that this compound would prevent the bortezomib-mediated increase of intracellular peptides. However, this was not the observed result. Instead, the combination of bortezomib and epoxomicin produced an increase in more peptides than that produced by either compound alone (Figure 5). These observations are difficult to explain through off-target effects of bortezomib; if bortezomib inhibits another peptidase that degrades the proteasome products, epoxomicin should reduce the production of the proteasome products and ultimately result in lower levels of peptides. But when all cells were exposed to bortezomib, the addition of epoxomicin resulted in higher levels of peptides (Figure 6). This could potentially be explained by the off-target effects of epoxomicin, although this compound is generally considered to be highly specific for the proteasome [21,58].

There is considerable diversity among proteasome forms, with variability of the regulatory caps, associated proteins, catalytically active beta subunits (e.g., β5 vs. β5i vs. β5t), and post-translational modifications [14]. Some of these variations have been found to have functional consequences in the ability of the proteasome to cleave certain substrates (ubiquitinated vs. non-ubiquitinated) or to affect the peptide products that are generated [14]. A few studies have compared the different forms for sensitivity to proteasome inhibitors. For example, bortezomib inhibits the β5 and β5i subunits with comparable potency, while carfilzomib is 5-fold more potent towards the β5 than the β5i subunit [59,60]. According to the GeoProfiles database, HEK293 cells express mainly the β5 subunit and very low levels of β5i or β5t (https://www.ncbi.nlm.nih.gov/geoprofiles). HEK293 cells also express very low levels of the β1i subunit, which has a cleavage specificity more similar to β5 than β1 subunits [61]. Based on their low levels of expression in HEK293 cells, it is not likely that β5i, β5t or β1i contribute to the production of the peptides identified in our present study. Each 20S proteasome core is able to bind multiple proteasome inhibitors, based on studies investigating the crystal structure of the 20S proteasome in complex with carfilzomib [59]. Based on this, it is likely that, in combination, bortezomib and epoxomicin can both access the same 20S core. Thus, it is possible that some of the β5 subunits in this core are blocked by bortezomib while other β5 subunits in the core are blocked by epoxomicin. However, because each inhibitor alone can block the proteasome, the combination should also block the cleavage of peptides at hydrophobic sites, and not lead to the observed increase of intracellular peptides.

The peptides detected in the present study vary in size from six to 59 amino acids, with a median length of 16 residues. Small peptides, <5 residues in length, are not readily detected by the mass spectrometry-based peptidomics method used in our study [30]. Therefore, it is not clear whether the absence of small peptides in the peptidomics data reflect a low abundance of these peptides in the cell extracts or the limitation of the mass spectrometry-based technique to detect small peptides. Taken together, our data fit with a model in which the proteasome cleaves proteins into intermediate-sized peptides and then further cleaves these into small peptides. If the latter step is more efficiently blocked by bortezomib than the initial steps, this would produce the observed increase in the levels of many intermediate-sized peptides. This could also account for the large increase in peptides derived from internal regions of the proteins, relative to those representing N- or C-terminal fragments (Figure 7). In theory, the vast majority of the proteasome-generated peptides should represent internal peptides, as is the case with proteomic studies that digest proteins with trypsin prior to mass spectrometry; there is only one N-terminal and one C-terminal peptide per protein, but many internal fragments [30]. Instead, approximately 30–50% of the observed peptides represent N- or C-terminal fragments in this study (Figure 7) and other studies [20]. Bortezomib increases the ratio of internal fragments relative to N-/C-terminal fragments (Figure 7, panels C and D) which is consistent with the scenario in which bortezomib blocks the further degradation of proteasome-generated peptides. Alternatively, epoxomicin and bortezomib have been reported to function as allosteric modulators that increase gate opening and this could potentially contribute to the observed results [62]. Further studies are necessary to better understand the exact mechanisms behind the increase in the levels of intracellular peptides caused by proteasome inhibitors.

Several studies have found that relative short-term treatments (e.g., minutes) with proteasome inhibitors affect cellular processes such as the consolidation of long-term potentiation [63] and neuronal-activity-induced calcium signaling [64]. While these effects may be due to changes in protein degradation, if the resulting peptides are biologically active, then the altered levels of these peptides may contribute to some of the observed effects of proteasome inhibitors. This idea, originally proposed in 2004 [6] and reviewed in 2010 [5] and 2019 [65], is supported by the recent finding that peptides released by the proteasome function in neuronal signaling [64]. Because proteasome inhibitors alter the levels of most intracellular peptides much more rapidly than the levels of most proteins, the short-term effects of proteasome inhibitors may be primarily due to the activity of the peptides. Such roles include altering protein–protein binding, protein trafficking, and protein folding [7].

## 4. Materials and Methods

### 4.1. Reagents

HEK293T cells were obtained from American Type Culture Collection. High glucose Dulbecco’s Modified Eagle’s Medium (DMEM), Dulbecco’s Phosphate Buffered Saline (DPBS) and penicillin/streptomycin were obtained from Invitrogen (Carlsbad, CA, USA) and fetal bovine serum (FBS) from VWR Life Science (Radnor, PA, USA). Hydroxylamine, glycine, sodium hydroxide, dibasic sodium phosphate and dimethyl sulfoxide (DMSO) were purchased from Sigma-Aldrich (St. Louis, MO, USA). Acetonitrile, hydrochloric acid, trifluoroacetic acid (TFA) mass-spectroscopy grade and C-18 spin columns were purchased from ThermoFisher Scientific (Waltham, MA, USA). Amicon Ultracel-10 centrifugal filter units were obtained from Millipore (Burlington, MA, USA). Other reagents and their commercial sources were b-AP15 (Boston Biochem, Cambridge MA, USA), cycloheximide (Sigma-Aldrich), bortezomib (LC Laboratories, Woburn MA, USA) and epoxomicin (Sigma-Aldrich). Anti-tubulin antibody was obtained from Sigma-Aldrich and anti-ubiquitin, anti-rabbit and anti-mouse antibodies were obtained from Cell Signaling Technology (Danvers, MA, USA). The isotopic labeling reagent, 4-trimethylammoniumbutyryl-N-hydroxysuccinimide (TMAB-NHS), containing either 0, 3, 6 or 9 atoms of deuterium (D0-, D3-, D6- and D9-TMAB-NHS, respectively) was synthesized as described previously [66].

### 4.2. Methods

#### 4.2.1. Treatment with b-AP15 and Western Blotting

HEK293T cells were grown in 6-well plates (35 mm culture dishes) to 80–90% confluence in DMEM supplemented with 10% FBS and penicillin/streptomycin. At the start of the experiment, the media was removed from the plates and cells were washed with DPBS (138 mM NaCl, 8.06 mM Na_2_HPO_4_, 2.67 mM KCl, 1.47 mM KH_2_PO_4_, 0.9 mM CaCl_2_ and 0.49 mM MgCl_2_). This was followed by serum-free media containing 1 µM b-AP15 and 0.05% DMDSO, or 0.05% DMSO alone as control. After 1 h of incubation, cells were washed twice with DPBS, extracted with 200 µL hot SDS-PAGE buffer, and boiled at 95 °C for 5 min. The lysates were centrifuged at 13,000× *g* for 5 min and SDS-PAGE was performed. After transfer to nitrocellulose, the blot was probed using an anti-ubiquitin antibody (1:1000) or an anti-α-tubulin antibody (1:5000) followed by an anti-rabbit antibody (1:2000) or anti-mouse antibody (1:2000) linked to horseradish peroxidase for detecting ubiquitin and α-tubulin, respectively. Blots were then incubated in enhanced chemiluminescence reagent and exposed to X-ray films.

#### 4.2.2. Large-Scale Cell Culture, Induction of Heat Shock, Treatment with B-AP15, Cycloheximide and Proteasome Inhibitors, and Peptide Extraction

HEK293T cells were grown to 80–90% confluence in 150 mm culture dishes, in the same media as described above. A single plate of cells was used for each group, and 2–4 replicates of each group were performed as described in Figures S1 and S2. At the start of the experiment, media were removed from the plates and cells were washed with DPBS. For heat shock, this was followed by the addition of serum-free media and incubation of two plates at 42 °C and two at 37 °C for either 20 or 60 min (see Figure S1). For the rest of the experiments, washing was followed by the addition of serum-free media containing the various compounds in 0.05% DMSO (1 µM b-AP15, 0.2 mM cycloheximide, combination of 0.5 µM bortezomib and 0.2 µM epoxomicin), or the appropriate control and incubation at 37 °C for 30–60 min (see Figures S1 and S2 for details). Media were removed, cells were washed with DPBS, detached from the plate by scraping into DPBS, and centrifuged at 800× *g* for 5 min. The wash buffer was supplemented with the appropriate compound at the same concentration as used for the treatment to ensure that enzyme inhibition was maintained during harvesting of cells. After centrifugation, the cell pellet was resuspended in 1 mL of 80 °C water and incubated in an 80 °C water bath for 20 min to inactivate proteases. This was followed by centrifugation (13,000× *g*, 30 min, 4 °C) and the sample was stored at −80 °C until peptide extraction.

For peptide extraction, the samples were thawed and centrifuged again, as above. The supernatant was cooled on ice and acidified with HCl to a final concentration of 10 mM. After 15 min incubation on ice, the lysate was centrifuged at 13,000× *g* for 30 min at 4 °C. The supernatant was removed and combined with 250 µL of dibasic sodium phosphate (0.4 M, pH 9.5). The mixture was stored at −80 °C until labeling.

#### 4.2.3. Isotopic Labeling and Mass Spectrometry

Quantitative peptidomics was performed using the differential isotopic labeling strategy with trimethylammonium butyrate (TMAB) activated with N-hydroxysuccinimide (NHS), as described [66]. Each group within an experiment was labeled with a different isotopic form of the tag, as indicated (Figures S1 and S2). The TMAB-NHS labels were dissolved in DMSO to a concentration of 400 µg/µL and 7.5 mg of label was used per plate of cells. At the beginning, the pH of the peptide extract was adjusted to 9.5 with 1 M NaOH. Labeling was performed over eight rounds; 2.3 µL of the label was added to the extract every 20 min. The pH was measured between each round and, if necessary, brought back to 9.5 for the first five rounds. For rounds 6–8, the pH was not adjusted after the addition of the TMAB-NHS reagent. After the final round of labeling, the pH was adjusted to 9.5 again and the extracts were incubated at room temperature for 90 min. Glycine (30 µL of 2.5 M) was added to quench any unreacted label. Following 40 min of incubation at room temperature, the labeled extracts for a single experiment were pooled and filtered through microfiltration units with a 10-kDa membrane (Amicon Ultracel-10). To ensure that only N-terminal amines and lysine side-chain amines of peptides are labeled with TMAB and not tyrosines, 30 µL of a 2 M solution of hydroxylamine hydrochloride was added over three rounds to the pooled and filtered sample to hydrolyze any labeled tyrosines. The pH was measured after the addition of hydroxylamine and adjusted to 9.0 with 1 M NaOH. The samples were desalted through C-18 spin columns and peptides were eluted using 160 μL of 0.5% TFA and 70% acetonitrile. Samples were freeze-dried in a vacuum centrifuge and stored at −80 °C until analysis by mass spectrometry.

Samples were resuspended in 10 μL of water and 2–5 μL were analyzed on a Synapt G2 mass spectrometer coupled to a nanoAcquity capillary liquid chromatography (LC) system (Waters, Milford, MA, USA). The peptide mixture was desalted online for three min at a flow rate of 5 μL/min of phase A (0.1% formic acid) using a Symmetry C18 trapping column (5 μm particles, 180 μm inner diameters, 20 mm in length; Waters). The mixture of trapped peptides was subsequently separated by elution with a gradient of 7–65% over 42 min of phase B (0.1% formic acid in acetonitrile) through a BEH 130 C18 column (1.7 μm particles, 75 μm inner diameter, 100 mm in length; Waters). The data were acquired in the data-dependent mode and the mass spectra of multiple-charged protonated peptides generated by electrospray ionization were acquired for 0.2 s from m/z 300–1600. The three most intense ions exceeding base peak intensity threshold of 2500 counts were automatically selected and tandem mass spectrometery (MS/MS) was performed by dissociation of the ions by 15 to 60 eV collisions with argon for 0.2 s. The typical LC and electrospray ionization conditions consisted of a flow rate of 250 nL/min, a capillary voltage of 3.0 kV, a block temperature of 70 °C, and a cone voltage of 50 V. The dynamic peak exclusion window was set to 90 s.

Spectra were analyzed using the MassLynx 4.0 software (Waters). Peak groups representing peptides labeled with different isotopic labels were identified and the relative intensity of each monoisotopic peak was used for further calculations. To quantify the relative levels of peptides, the peak intensity of each treated group was compared to the average of the control replicates in each experiment. To identify peptides, MS/MS data were analyzed using the Mascot search engine (Matrix Science Ltd., London, UK) and the IPI_human data base (91,464 sequences; 36,355,611 residues). No cleavage site was specified. Modifications included the TMAB labels (termed ‘GIST’ in Mascot) and also N-terminal protein acetylation, methionine oxidation, and cyanylation of Cys. Results were manually interpreted to eliminate false positives, using previously described criteria [66,67].

## Supplementary Materials

The following are available online at https://www.mdpi.com/2218-273X/9/6/207/s1, Figure S1 and Figure S2; Table S1–S4.
