# Supplementary figures and images for: Effect of Protein Denaturation and Enzyme Inhibitors on Proteasomal-Mediated Production of Peptides in Human Embryonic Kidney Cells
Source: Biomolecules. 2019 May 28;9(6):207. doi: 10.3390/biom9060207 (PMC6627375; doi:10.3390/biom9060207)

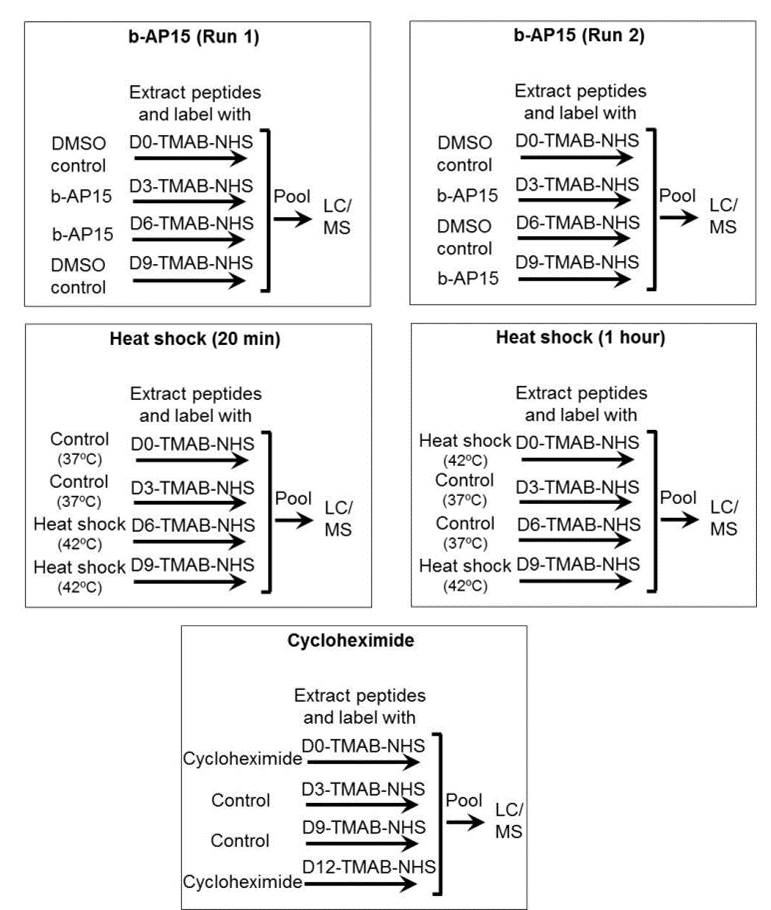

Supplement: Supplementary file 1 [file biomolecules-09-00207-s001.zip › 2 round supplementary/S1 Figure - 04-01-2019.tif]

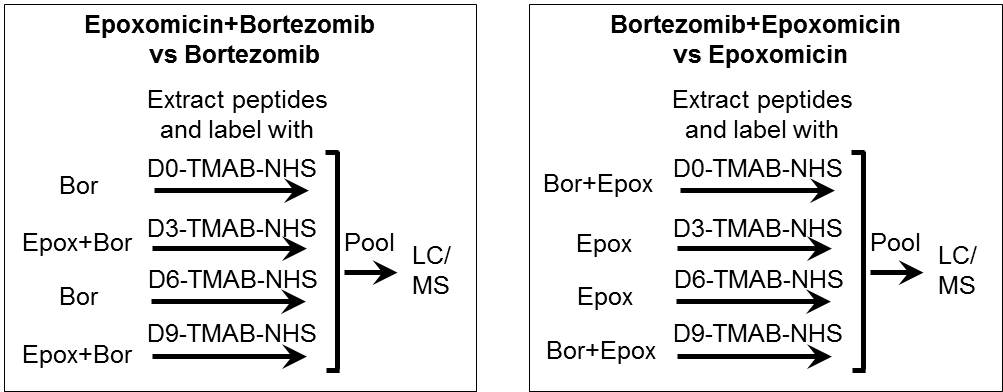

Supplement: Supplementary file 1 [file biomolecules-09-00207-s001.zip › 2 round supplementary/S2 Figure - 04-01-2019.tif]
